# Correlation of cochlear aperture stenosis with cochlear nerve deficiency in congenital unilateral hearing loss and prognostic relevance for cochlear implantation

**DOI:** 10.1038/s41598-021-82818-9

**Published:** 2021-02-08

**Authors:** Eva Orzan, Giulia Pizzamiglio, Massimo Gregori, Raffaella Marchi, Lucio Torelli, Enrico Muzzi

**Affiliations:** 1 Otorhinolaryngology and Audiology, Institute for Maternal and Child Health IRCCS “Burlo Garofolo”, Trieste, Italy; 2 Radiology, Institute for Maternal and Child Health IRCCS “Burlo Garofolo”, Trieste, Italy; 3 Department of Medicine, Surgery and Health Sciences, University of Trieste, Trieste, Italy

**Keywords:** Anatomy, Pathogenesis

## Abstract

The use of neonatal hearing screening has enabled the identification of congenital unilateral sensorineural hearing loss (USNHL) immediately after birth, and today there are several intervention options available to minimize potential adverse effects of this disease, including cochlear implantation. This study aims to analyze the characteristics of the inner ear of a homogeneous group of congenital non-syndromic USNHL to highlight the features of the inner ear, which can help in clinical, surgical, and rehabilitative decision-making. A retrospective chart review was carried out at a tertiary referral center. Systematic diagnostic work-up and rigorous inclusion–exclusion criteria were applied to 126 children with unilateral hearing impairment, leading to a selection of 39 strictly congenital and non-syndromic USNHL cases, undergoing computed tomography (CT) and magnetic resonance (MR) imaging studies. The frequency and type of malformations of the inner ear in USNHL and unaffected contralateral ears were assessed, with an in-depth analysis of the deficiency of the cochlear nerve (CND), the internal auditory canal (IAC) and the cochlear aperture (CA). Inner ear anomalies were found in 18 out of 39 (46%) of the USNHL patients. In 1 subject, the anomalies were bilateral, and the CND resulted in the predominant identified defect (78% of our abnormal case series), frequently associated with CA stenosis. Only 3 out of 14 children with CND presented stenosis of the IAC. CND and CA stenosis (and to a much lesser extent IAC stenosis) are a frequent association within congenital and non-syndromic USNHL that could represent a distinct pathological entity affecting otherwise healthy infants. In the context of a diagnostic work-up, the evaluation with CT and MRI measurements should take place in a shared decision-making setting with thorough counseling. Both imaging techniques have proven useful in differentiating the cases that will most likely benefit from the cochlear implant, from those with potentially poor implant performance.

## Introduction

Estimates of the universal newborn hearing screening programs suggest that around 1–2 in 1000 infants have significant hearing loss, with a unilateral deficit representing one-third of all the children born with permanent hearing loss^1^. Interest in the management of childhood unilateral sensorineural hearing loss (USNHL) has significantly increased in recent years. The reasons stem primarily from the growing awareness that functional binaural hearing requires sound input for both ears^2^. Furthermore, thanks to the widely adopted universal newborn hearing screening programs, the diagnosis of congenital USNHL can now be made in the first few months of life instead of being detected in preschool and school-age^3,4^, allowing early treatment. However, another incentive for USNHL treatment arises from the technological advancements of hearing devices^5,6^.

Gordon and Papsin (2019) described the binaural hearing as a function that provides a map of our environment in all the directions, which we use to separate and group auditory streams^7^. Adults with USNHL experience difficulties in localizing sounds and understanding speech, competing with environmental noise^8^. If the USNHL is congenital or occurs in the first years of life, the localization skills seem to be less compromised, possibly due to the successful use of monoaural cues through developmental compensation^9^. However, many papers highlighted the negative consequences of USNHL on children’s development, which include delayed or impaired auditory skills^10^, increased risk of receptive and expressive language problems^11^, behavioral and educational challenges^12^, and reduced hearing-related quality of life^13^. The etiology of congenital USNHL is much less defined than bilateral sensorineural hearing loss, and although specific causative agents are not often recognized^14,15^, the literature indicates a high frequency of inner ear malformations in congenital USNHL cases^16–18^. Temporal bone anomalies are identified in 29–40% of pediatric USNHL when assessed by CT. In 10–25% of the cases, MR detects anomalies of the cochlear, vestibular, or facial nerves, of the cerebellopontine angle structures, or auditory pathways^16,19^. It is essential to recognize an inner ear malformation to clarify the underlying pathology, to offer critical information in terms of possible hearing loss progression or contralateral involvement, as well as to provide tips for counseling and treatment. Indeed, rehabilitation management has been variably provided to children with USNHL, with a range of options available but, so far, no evidence of the superiority of one approach to another^20^. In the past, the most conventional approach to congenital USNHL was watchful waiting and monitoring of auditory-language development^21^. There are now several devices available for USNHL prosthetic treatment: acoustic hearing aids, contralateral routing of signal hearing aids, bone conduction hearing implants and cochlear implants^22^. Cochlear implant has recently sparked increasing interest as a treatment option^23,24^, described as the most likely tool for improving binaural hearing for adults and children affected by severe-to-profound USNHL^25^. A systematic review established that no firm conclusions can be drawn on the efficacy of cochlear implant in children with USNHL, due to the heterogeneous findings and small sample sizes^26^. The significant variability of the outcomes can be attributed to heterogeneous etiologies and times of the onset of USNHL (i.e., congenital and acquired, with or without hearing experience or auditory deprivation^27^).

This study aims to analyze the characteristics of the inner ear of a homogeneous group of congenital non-syndromic USNHL in order to highlight the anatomical features that can help in clinical, surgical, and rehabilitative decision-making. We focused on:

- Characteristics of inner ear malformations on the affected side;
- Anomalies on the contralateral unaffected side;
- Cochlear nerve deficiency (CND), cochlear aperture (CA) stenosis and internal auditory canal (IAC) stenosis.

## Material and methods

### Subjects

This retrospective chart review involves all children who have been diagnosed with a permanent USNHL and assessed using imaging techniques from 2012 to 2019 at the Department of Otorhinolaryngology and Audiology of the Institute for Maternal and Child Health IRCCS “Burlo Garofolo”, Trieste, Italy. USNHL was defined as bone conduction pure tone threshold average of 500, 1000 and 2000 Hz, > 40 dB hearing level (HL) in the affected ear and < 15 dB HL in the contralateral unaffected ear. Hearing loss degree was classified as moderate (41–60 dB HL), severe (61–80 dB HL) or profound (> 80 dB HL). The identified children (n = 126) underwent a systematic diagnostic work-up, which included ear, nose and throat examination, ophthalmologic evaluation, kidney ultrasonography, electrocardiogram, and inner ear imaging, mainly aimed at verifying the presence of syndromes or inner ear malformations. Cytomegalovirus infection was ruled out after the failure of the universal newborn hearing screening. In the case of older infants with late USNHL diagnostic work-up, a detailed brain MR review was performed, to highlight abnormalities possibly involved with a congenital cytomegalovirus infection syndrome. Audiological tests confirmed a hearing loss and classified its type, laterality and degree. In order to gather a homogeneous population of congenital USNHL, strict inclusion and exclusion criteria have been applied (see Table 1).

**Table 1:** Inclusion and exclusion criteria applied in the study.

Inclusion criteria	Exclusion criteria
Preverbal USNHL of significant degree (PTA^0.5–1–2 kHz^ ≥ 40 dB HL)	Contralateral hearing exceeding normal threshold values (PTA^0.5–1–2 kHz^ > 15 dB HL)
No hearing risk factor for post-natal or acquired onset (i.e. cytomegalovirus infection, neonatal intensive care unit admittance, trauma, meningitis etc.)	Sudden USNHL onset and/or unknown neonatal history and pathological medical history
Isolated USNHL (i.e. no craniofacial malformation or other syndrome associations; no CNS findings known to correlate with acquired sensorineural hearing loss)	Uncompleted documentation regarding clinical and instrumental work-up
Temporal bone CT and cerebral MR imaging	Uncompleted CT and/or MR performance

Thirty-nine children (20 males, 19 females) who met the inclusion and exclusion criteria, as reported in Table 1, were included for the analysis. The mean age at diagnosis was 4.4 years (3.4 standard deviation); 11 out of 39 subjects underwent the universal newborn hearing screening. USNHL was present on the left side in 27 children (69.2%) and on the right side in 12 children (30.8%). The hearing loss’ degree was profound in 29 out of 39 cases, severe in 5 cases and moderate in the last 5 cases.

### Imaging evaluation

Both temporal bone CT and cerebral MR were performed in the same session during natural sleep or sedation, if necessary. Temporal bone CT: patients were examined by high-resolution CT Philips Brilliance 40, with a slice thickness of 0.67 mm, slice increment of 0.33 mm, collimation 16 × 0.625 mm, time of rotation 1 s., amperage 140 kV, mAs 400mAs, CTDI 93.7 mGy, DLP mGy*cm 503.16, the scale of the length of 52.8 mm and field of view of 155 mm. Temporal bone MR: patients were examined with Philips Ingenia 1.5 T with DRIVE 3D HR sequences: TE 198 ms; TR 1500 ms; ETL 40; the field of view of 160 mm; isotropic voxel of 0.6 × 0.6 × 0.6 mm, interpolate 50%; matr. 252 × 265; scan percentage 80%; slice thickness 0.9 mm.

The measurements were reconstructed on axial, coronal, and oblique-sagittal views using the Picture Archiving and Communication System. The measures were reported in millimeters; dimensions from 1 to 6 were measured on axial, while 8–10 on the coronal plane. The standard range values for the basal and middle turn of the cochlea, the cochlear height, the bone island width of the semicircular canals, and the coronal width of the internal auditory canal were calculated based on the average measurement of our normal controls minus/plus 2 standard deviations-SD^28^. The average range values were directly selected from the previously published literature for other parameters such as the cochlear aperture, vestibular aqueduct, cochlear length, cochlear nerve^29–32^. Precisely, this study was based on the following parameters and standard range measurement:

1. The cochlear aperture (CA) was measured in its mid-portion at a mid-modiolar level and was classified as “normal”, “aplastic” (when completely ossified) or “stenotic” (≤ 1.2 mm), based on Lim et al.^31^ who provided a cut off elaborated with an explicit methodology;
2. The cochlear basal turn was measured from the bone cochlea near the oval window to the furthest end of the basal turn (range: mean value in normal controls ± 2 SD; 8.1–9.7 mm)^28^;
3. The cochlear middle turn was measured in its largest part (range: mean value in normal controls ± 2 SD; 3.8–4.6 mm)^28^;
4. The cochlear height was measured from the base of the modiolus, through the modiolus, to the cochlear apex^30^ (range: mean value in normal controls ± 2 SD; 3.8–5 mm)^28^;
5. The vestibular aqueduct was measured at the midpoint between the common crus and the operculum^32^. It has been defined “enlarged” if greater than 2 mm;
6. The bony island width of the posterior semicircular canal was measured in axial view at its greatest diameter (range: mean value in normal controls ± 2 SD; 4.3–6.3 mm)^28^;
7. The bony island width of the lateral semicircular canal was measured in coronal view at its greatest diameter (range: mean value in normal controls ± 2 SD; 2.4–4.8 mm)^28^;
8. The bony island width of the superior semicircular canal was measured in coronal view at its greatest diameter (range: mean value in normal controls ± 2 SD; 4.4–6 mm)^28^;
9. The cochlear length was measured from the medial point of the round window (air-perilymph interface) to the more external bony point, passing through the modiolus (range: 8.1–9.59 mm)^30^;
10. The coronal width of the internal auditory canal (IAC) was drawn perpendicular to an imaginary line from the transverse crest to the mid-point of the porus. (range: mean value in normal controls ± 2 SD; 3.9–6.7 mm)^28^.
  MR measurements:
11. The cochlear nerve was evaluated on axial and especially on sagittal-oblique images. It was classified as “aplastic,” “hypoplastic,” or “normal” according to its size compared to the ipsilateral facial nerve^29^ and contralateral cochlear nerve diameter. The terms of aplasia and hypoplasia are also referred as cochlear nerve deficiency (CND);
12. The endolymphatic sac was defined as “not visible” or “visible,” although not included in the statistical comparison.
  A dedicated pediatric radiologist, a temporal bone imaging expert, with no information on the affected child's ear, examined and compared the imaging.

Inner ear malformations were documented according to Sennaroğlu Classification^33^.

### Normal controls

We evaluated 14 children (23 ears) not affected by sensorineural hearing loss who performed audiometry, CT and MR for other reasons, mainly of chronic ear surgery. In the case of cholesteatoma history, the affected ear was ruled out for the possible presence of cochleovestibular anomalies in this type of pathology^34^.

### Statistics

Given the small group of subjects and the non-normal distribution of the data samples (Shapiro–Wilk normality tests, *p* < 0.05), we decided to obtain a statistical indication using a nonparametric test. We described variables with medians and quartiles. Comparisons between groups were made using Wilcoxon rank-sum test for continuous variables, and Fisher’s exact test for categorical variables. *P* values < 0.05 were considered significant. Data were analyzed and visualized using Excel (version 16.23) and R Commander (version 3.6.0 GUI 1.70)^35^.

### Ethical approval

The study was conducted according to the 1964 Helsinki declaration and its later amendments, under the framework of the research project 17/17 approved by the institutional review board and by the Italian Ministry of Health-CCM (project 12/2013).

### Informed consent

Informed consent was obtained from a parent and/or legal guardian of all participants included in the study.

## Results

Table 2 summarizes the identified anomalies with the associated hearing loss degree. Of 39 cases analyzed, 18 (46%) had an inner ear malformation on the USNHL side with several overlapping conditions. The only case with bilateral malformation presented a defective apical part of the modiolus on both sides (similarly to the incomplete partition type 2, IP2, but without the enlarged vestibular aqueduct, EVA), with a milder expression on the normal hearing side.

**Table 2:** The table summarizes the type of anomaly, the side and the degree of hearing loss of the 18 patients with inner ear malformations.

Type of anomaly	No	Side (No.)	Hearing loss degree (No.)
Hypoplasia of the cochlear nerve (CN)	7	Left (7)	Moderate (1) Severe (3) Profound (3)
Aplasia of the cochlear nerve (CN)	7	Left (5) Right (2)	Profound (7)
Cochlear aperture (CA) stenosis (≤ 1.2 mm)	10	Left (9) Right (1)	Severe (1) Profound (9)
Internal auditory canal (IAC) stenosis (< 3.9 mm)	3	Left (2) Right (1)	Profound (3)
Enlarged vestibular aqueduct (EVA) (> 2 mm)	6	Left (3) Right (3)	Moderate (1) Severe (2) Profound (3)
Incomplete partition (IP)	1 (IP-1)	Left (1)	1 Severe (1)
	1 (IP-2)	Right (1)	1 Profound (1)

Hypoplasia/aplasia of the cochlear nerve and cochlear aperture stenosis were the most frequent overlapping conditions (10 cases).

The CND, classified as hypoplasia (n = 7) or aplasia (n = 7), was the more represented pathological picture (35.9%). All aplasia cases showed a profound hearing loss, while hypoplasia was associated with a variable degree of hearing loss. Two CNDs were found in the right ear, while the other 12 CND cases belonged to left ears. In 10 out of 14 USNHL cases, the CND was associated with CA stenosis (Fisher’s exact test, *p* = 0.001), and in only 3 CNDs a stenotic IAC was found. The 4 CNDs without CA stenosis were associated with an EVA in 2 cases, with an IP2 in 1 case, while the last CND case (cochlear nerve aplasia) presented only with an isolated stenotic IAC.

**Figure 1:**
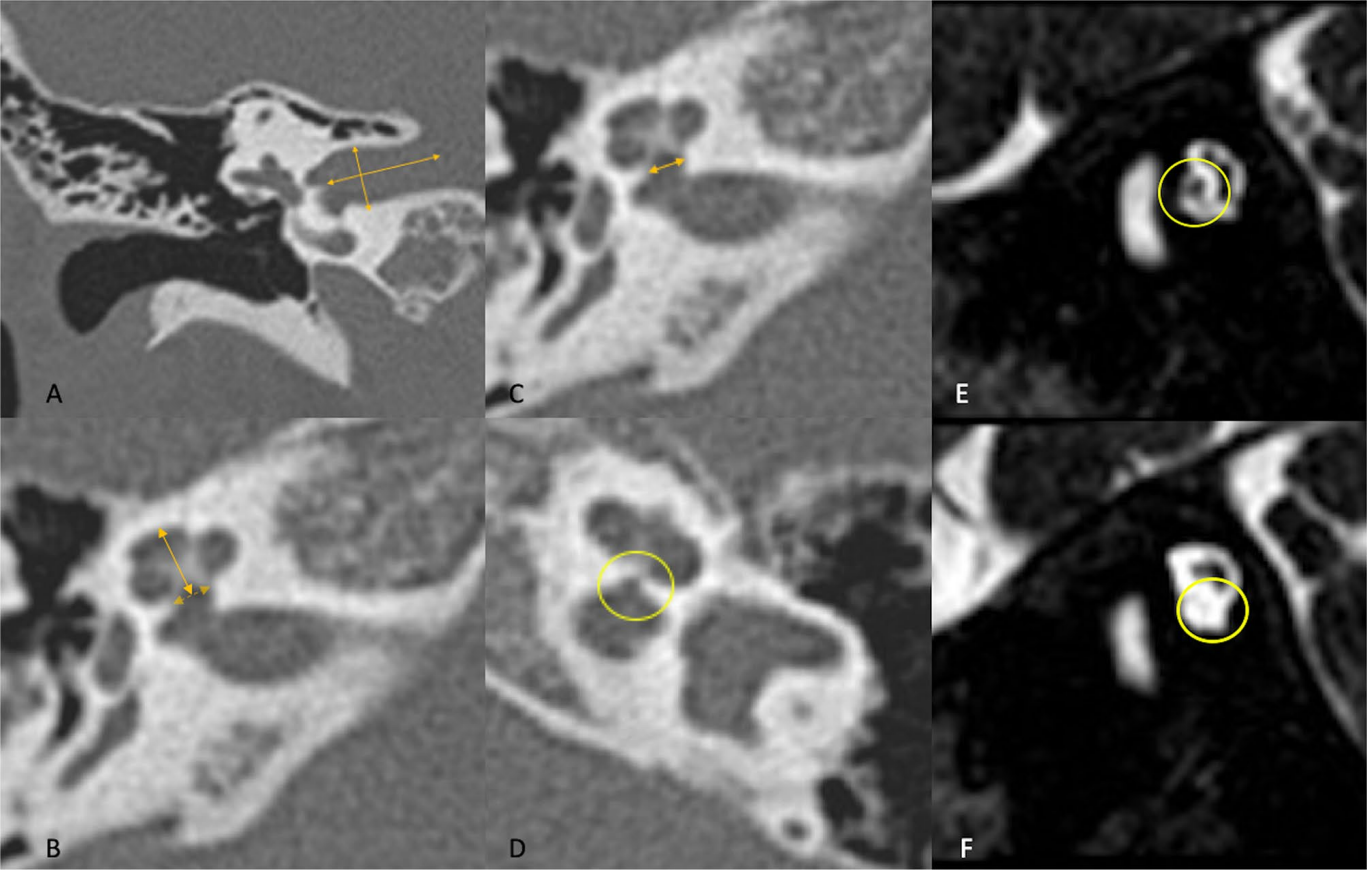
Inner ear CT scans with measures of inner auditory canal, cochlear height, cochlear aperture, and MR images of cochlear nerve. (**A**) Inner auditory canal (CT coronal view, right ear, normal); (**B**) Cochlear height (CT axial view, right ear, normal); (**C**) Cochlear aperture (CT axial view, right ear, normal); (**D**) Severe cochlear aperture stenosis (CT axial view, left ear); (**E**) Normal cochlear nerve, the bony canal is well visible and comparable with the facial nerve bony canal; (MR oblique-sagittal view); (**F**) Hypoplastic cochlear nerve, the bony canal is almost completely ossified, (MR oblique-sagittal view).

Examples of inner ear CT scans with measures of inner auditory canal, cochlear height, cochlear aperture, and MR images of cochlear nerve are provided in Fig. 1.

A total of 6 EVAs were identified: apart from the 2 already mentioned as associated with CND, 3 EVAs were isolated, 1 was associated with IP type 1 malformation (infrequent association). In 4 out of 6 EVAs, the endolymphatic sac was visible on MR images. In 1 case, it was possible to visualize the endolymphatic sac without the appreciation of a vestibular aqueduct diameter > 2 mm.

The anomalies of the bony island of the lateral semicircular canal (SCC) did not appear to be related explicitly to USNHL: we detected reduced bony island dimensions in 3 unaffected ears and 2 USNHL sides. Only in one subject of our series, the lateral SCC bony island anomaly was bilateral: the USNHL side harbored the IP 1 malformation with lack of lateral SCC bony island, and the contralateral normal hearing side presented a lateral SCC bony island < 2.4 mm.

**Figure 2:**
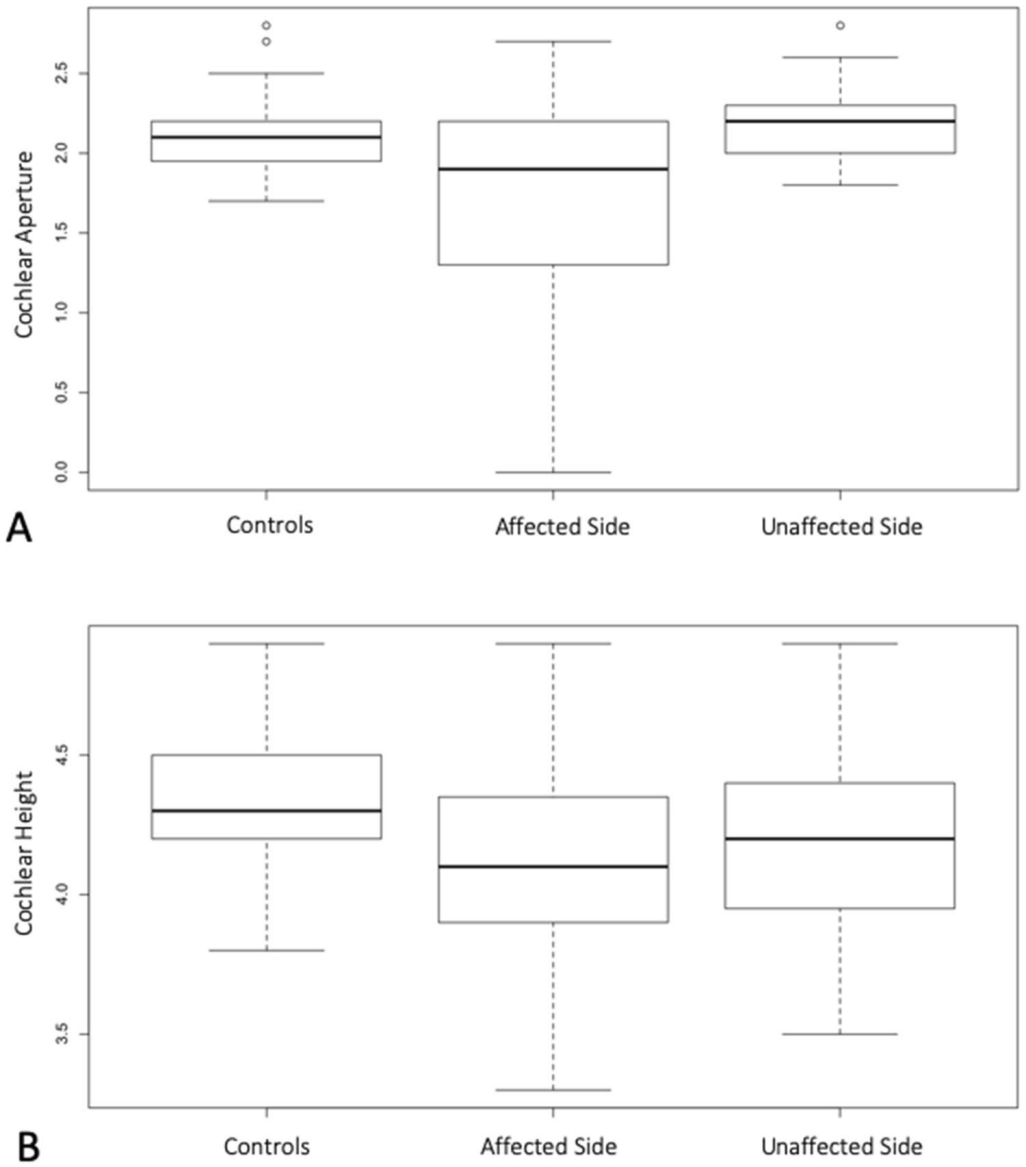
The box-plot A represents the comparisons of the cochlear aperture’s dimension in millimeters between normal controls, affected side, and unaffected side. The box-plot B represents the comparison of the cochlear height’s dimensions between the same groups. In the y-axis, the values represent the *measurement* in millimeters^35^.

Statistical comparisons of the measured parameters are presented in Table 3: the only significant differences between affected and unaffected sides of USNHL subjects and normal controls regards the CA and the cochlear height dimensions. As also represented in Fig. 2, the CA showed a significantly smaller dimension in the affected side in comparison to both the contralateral side (*W* = *438.5, p* < *0.01*) and normal controls (*W* = *587, p* = *0.05*). Overall, also cochlear height measurements were bilaterally smaller in USNHL children, even though only 3 out of 39 in the affected side and 4 in the unaffected side subjects resulted below our normal range values. (All measured parameters values resulting outside the normal range can be viewed in the Supplementary Table S1 online).

**Table 3:** Median and quartiles (q1; q3) of the measurements acquired from computed tomography and group differences (bold, *p* < 0,05).

Parameter	Affected side Group 1	Unaffected side Group 2	Normal controls Group 3	Wilcoxon rank sum test with continuity correction* Group 1 versus Group 2	Wilcoxon rank sum test with continuity correction* Group 1 versus Group 3	Wilcoxon rank sum test with continuity correction* Group 2 versus Group 3
Cochlear Aperture	1.9 (1.3; 2.2)	2.2 (2.0; 2.3)	2,1 (2.0; 2.2)	***W = 438.5 p < 0.01***	***W = 587 p = 0.05***	*W* = *390.5* *p* = 0.79
Basal turn	8.8 (8.3; 9.2)	8.7 (8.4; 9.0)	9.0 (8.6; 9.2)	*W* = *848.5* *p* = 0.82	*W* = *506.5* *p* = 0.61	*W* = *588* *p* = 0.08
Middle turn	4.2 (4.1; 4.6)	4.2 (4.1; 4.4)	4.2 (4.0; 4.3)	*W* = *756* *p* = 1.00	*W* = *376* *p* = 0.73	*W* = *378* *p* = 0.61
Cochlear height	4.1(3.9; 4.4)	4.2 (4; 4.4)	4.3 (4.2; 4.5)	*W* = *687.5* *p* = 0.93	***W = 627 p < 0.01***	***W = 612 p = 0.03***
Cochlear length	8.9 (8.6; 9.3)	8.9 (8.5; 9.2)	9.0 (8.6; 9.7)	*W* = *851.5* *p* = 0.73	*W* = *426* *p* = 1.00	*W* = *537* *p* = 0.40
Coronal width of the IAC	5 (4.6; 6.1)	5.3 (4.8; 6.2)	5.3 (4.8; 5.7)	*W* = *627* *p* = 0.37	*W* = *482* *p* = 1.00	*W* = *404.5* *p* = 1.00
Lateral SCC	3.4 (3.1; 3.8)	3.3 (3.2; 3.8)	3.8 (3.5; 4.0)	*W* = *776* *p* = 1.00	*W* = *558* *p* = 0.14	*W* = *572.5* *p* = 0.14
Superior SCC	5.1 (4.8; 5.4)	5.1 (4.6; 5.4)	5.3 (4.9; 5.5)	*W* = *819* *p* = 1.00	*W* = *502* *p* = 0.67	*W* = *557.5* *p* = 0.22
Posterior SCC	5.1 (4.6; 5.6)	5.2 (4.7; 5.6)	5.4 (4.9; 5.7)	*W* = *733* *p* = 1.00	*W* = *507.5* *p* = 0.59	*W* = *525.5* *p* = 0.53

*SCC* semicircular canals, *IAC* inner auditory canal. *The *p* values were corrected for the multiple comparisons with the Bonferroni correction method.

**Figure 3:**
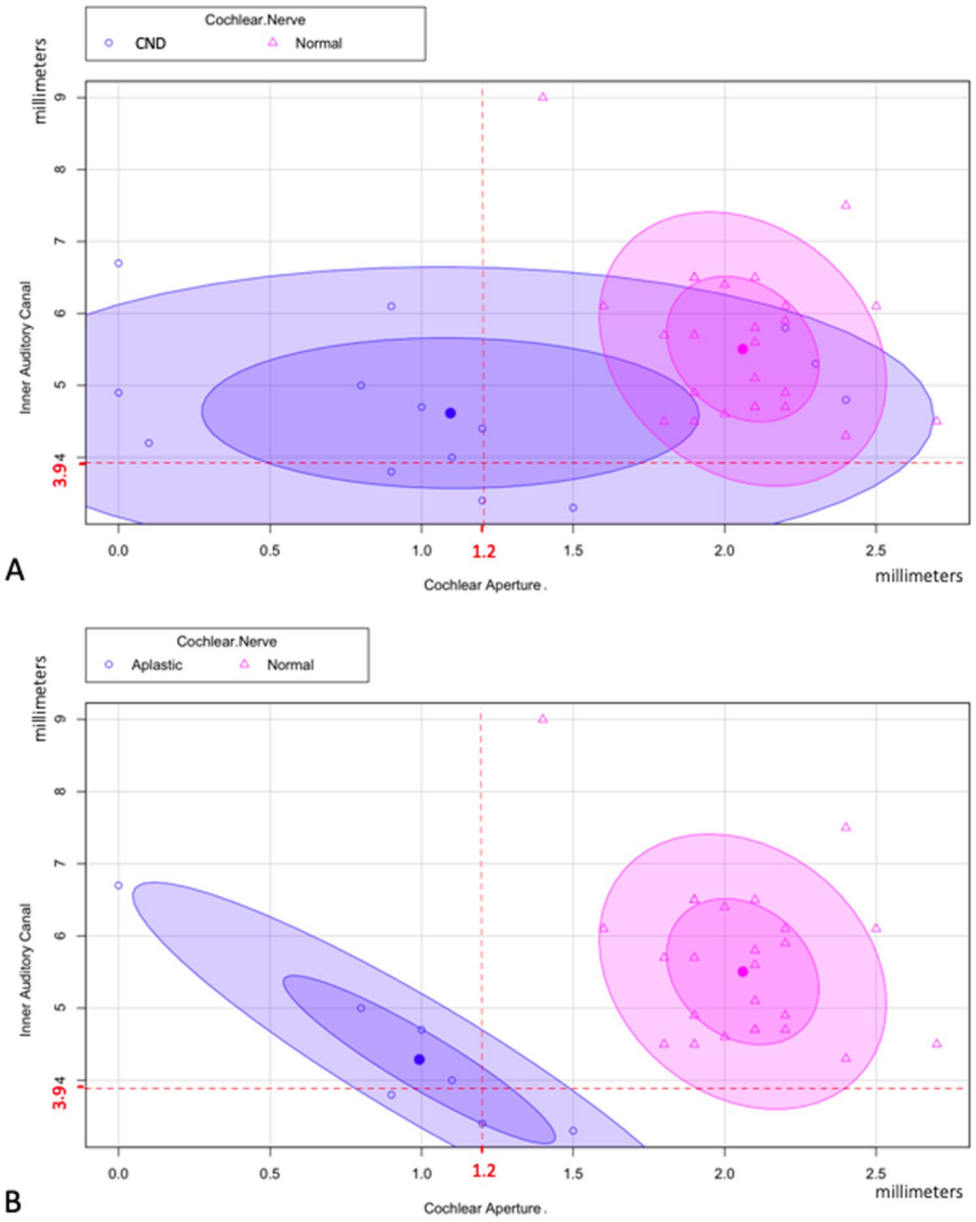
Dispersion graphs^35^ of IAC and CA measures, indicating that the size of CA is a more accurate indicator of CND, especially in case of cochlear nerve aplasia. **A**. Comparison between the affected ears with CND (aplasia and hypoplasia, 14 cases, blue area) and affected ears with normal cochlear nerve (25 cases, pink area). CA measures are represented on x-axis, while the IAC measures are represented in the y-axis. The cut offs to define the IAC or CA stenosis are indicated in red. The central point of the blue area represents the average dimensions of IAC and CA in patients with CND (IAC = 4.7 mm, SD = 1.2; CA = 1.2 mm, SD = 0.7), while the central point of the pink area represents the average dimensions of the same parameters but in patients with normal CN (IAC = 5.6 mm, SD = 1.1; CA = 2.1 mm, SD = 0.7). For both areas, the internal ellipse represents 1 standard deviation, while the external one represents 2 standard deviations. **B**. The blue area focuses on CND cases with nerve aplasia^7^, after exclusion of hypoplasia cases (central point: IAC = 4.4 mm, SD = 1.1; CA = 0.9 mm, SD = 0.7).

Finally, the dispersion graphs on Fig. 3 compare CND ears^14^ with ears that present a normal cochlear nerve^25^. The Fig. 3A highlights that ears with CND have lower CA dimension compared with ears without CND. This difference is more evident when the CND is restricted to only aplasia cases (Fig. 3B).

## Discussion

Our study confirms that individuals with congenital USNHL are more likely to have inner ear malformations than those with congenital bilateral hearing loss^36^. Anomalies are rarely present on both sides, only in 1 case of our series. A CND characterizes a large portion (35.9%) of our population, most frequently associated with CA stenosis. Both of these anomalies have been previously reported in the literature, although more often individually, or described as a possible feature within more heterogeneous populations^33,37,38^.

Unlike most studies describing inner ear anomalies in USNHL patients^38–41^, we adopted strict exclusion and inclusion criteria to focus on a strictly selected population. Besides, only a few reports have analyzed USNHL functionality with a double imaging technique (CT and MRI) on a significant number of patients with non-syndromic congenital USNHL. A multi-institutional retrospective imaging study review was conducted by Shah et al.^39^ on 219 patients with USNHL aged 0–18 years that underwent CT and/or MRI. The overall prevalence of abnormal radiological findings associated with USNHL was 40%. EVA was the most common positive finding (15%) identified on CT imaging, while CND was the most common anomaly (52%) in 16 out of 31 patients who underwent MRI only. Similarly, to our description, there were many patients with more than one positive finding on imaging. This study did not include a measurement of the CA on CT images, and therefore it was not possible to relate CA stenosis and CND in these patients. By applying stricter inclusion criteria, Lim et al.^31^ analyzed the medical records and temporal bone CT scans of 42 patients under the age of 13 years with a diagnosis of USNHL with unknown etiology (ototoxic, sudden hearing loss, trauma, ear infection, syndromes, craniofacial anomalies, ear surgery were excluded). A CA < 1.2 mm was detected in 52.4% of the total population. Even this study did not include MR images nor a normal control group. Vilchez-Madrigal et al.^42^ compared the CT imaging studies of 36 children with unilateral CA stenosis (< 1.0 mm), with controls without temporal bone injury. Contralateral ears had smaller CA (*p* < 0.00) and cochlea (*p* < 0.00) than controls, although to a lesser extent than on the stenotic side.

Thanks to the comprehensive anatomical imaging study of the inner ear structures in a homogeneous population, our work has linked CA stenosis and CND as a specific and frequent pathological profile in congenital non-syndromic USNHL. Contrary to general clinical opinion, aplasia/hypoplasia is not systemically or frequently associated with IAC stenosis in congenital USNHL. Only 2 cases of aplasia out of 14 CND showed the co-presence of a stenotic CA and an IAC stenosis, and only in 1 case out of 14 CND the stenotic IAC was isolated. As indicated by Tahir et al.^37^, the detection of IAC stenosis alone may be insufficient to indicate a CND: therefore, the CA is also required to predict cochlear nerve status. It is worth observing that there is no general agreement regarding how to measure the IAC width^37^, and the CA’s diameter can therefore represent a more reliable measure. Further morphological and genetic studies could, in the future, define whether the heterogeneity of the presence of the three anomalies described (CND, CA stenosis, and IAC stenosis) belongs to a continuum of the pathology of the inner ear.

The interest in CA is aroused by the fact that a narrow cochlear aperture probably indicates an anatomical or functional deficiency in the cochlear nerve. Consequently, a CA stenosis appeared to be a criterion for a negative outcome of the cochlear implant surgery, indicating that patients with stenotic CA demonstrate poorer cochlear implants results than patients with normal CA. Chung et al.^43^ compared the post-cochlear implant speech performances among 3 groups of various CA diameters (< 1.4 mm; 1.4–2.0 mm; > 2.0 mm). CND occurred more frequently in the group with a diameter of < 1.4 mm which, also showed a worse post-cochlear implant outcome.

The observations on the imaging of our work are relevant in today's perspective of the audiological management of congenital USNHL. Today's advantage in identification times and observations of our study stress the importance of adopting a very comprehensive diagnostic work-up in order to characterize the USNHL critical elements that can lead to the most rational and timely therapeutic approach.

The literature has discussed the need for early imaging in USNHL^44^. The main issue is exposure to CT radiations. We believe that a comprehensive imaging evaluation should take place in a shared decision-making setting with thorough counseling, taking into account that inner ear malformations are associated with congenital USNHL and an increased risk of developing bacterial meningitis in the pediatric population^17^. This was the case of one child in our series, who presented a cochleovestibular cystic malformation (IP-1), known to be associated with a higher risk of spontaneous cerebrospinal fluid fistula and recurrent meningitis^33^. An early temporal bone CT in the diagnosis of USNHL could be 17.4 to 80.3 times more likely to help prevent meningitis than to be associated with a subsequent malignant abnormality due to exposure to CT radiations^18^.

Interventions in pediatric USNHL include surgical and non-surgical devices that can be divided into 2 broad categories: in one approach, the intent is to transmit auditory stimuli that come from the impaired side to the healthy ear^45^; the second, aims to recover binaural hearing through traditional amplification or cochlear implantation. Contralateral routing of signal hearing aid, bone conduction hearing implants or cochlear implants are indicated for USNHL in the range of single-sided deafness with the abovementioned differential approach: contralateral routing of signal hearing aid and the bone conduction hearing implant deliver the stimuli to the normal hearing ear, while the only chance to restore a real binaural hearing in single side deafness is a cochlear implant. Bone conduction hearing implant can offer more advantages than contralateral routing of signal hearing aid amplification for USNHL^46^. However, there is still no compelling evidence that one of these strategies is superior to others in terms of hearing outcomes and cost-utility^47,48^. As regards the pediatric population, studies of cochlear implant results frequently include non-congenital USNHLs, i.e., children with some binaural hearing experience and short hearing deprivation^27^. Cochlear implants are still to be considered an emerging treatment option for children with congenital USNHL, and even the most recent literature reports only limited descriptions of the benefit of cochlear implant in these cases^20,49^. Therefore, evidence-based decisions regarding the most appropriate management in USNHL children are still lacking. In this perspective, our study shows that over a third of congenital and non-syndromic "idiopathic" USNHLs affecting otherwise healthy infants may not be suitable for current cochlear implant technology. In fact, among other factors known to influence cochlear implant outcomes, the choice (and benefits) of a cochlear implant depends on neuronal health and the presence of an inner ear malformation. Aplasia or hypoplasia of the auditory nerve is the primary concern, due to the possibility of compromising nerve stimulation through electrical impulses delivered by the cochlear implant. The literature reports that children with CND are generally considered poor candidates for cochlear implant surgery, and only a minority of children implanted with CND achieve good results^50^.

The present study has some limitations. The retrospective nature of data collection makes it subject to possible bias. Despite the strict inclusion and exclusion criteria, it is sometimes difficult to ascertain the congenital origin of the USNHL. Only 11 of the 39 USNHL children investigated were detected through the UNHS program, which became mandatory in our region in 2012, and in Italy, in 2017. Similarly, although all children performed a brain MR whose analysis excluded subjects with signs of congenital cytomegalovirus infection syndrome^51^, it is only with the recent introduction of cytomegalovirus testing on urine samples of infants in their first weeks of life that is possible to exclude a congenital cytomegalovirus -related hearing loss^52^.

Another issue is the cochlear nerve measurements based on comparison with a neighboring nerve rather than a range of reference values of the nerve diameter. Furthermore, the non-visualization of cochlear nerve in MR does not necessarily imply the complete absence of nerve fibers due to the difficulty of detecting small nerves^53^. The visibility of the nerve depends on the intensity of the magnetic field. The hypoplasia/aplasia ratio would increase if conducted using a device with higher magnetic field. Future prospective studies together with an accurate diagnostic work-up and strict imaging criteria will allow obtaining more details on the prevalence and clinical distinction of the different pathogenetic profiles in congenital USNHL. However, this study’s strength is to rely on a blind assessment of inner ear imaging performed by a dedicated radiologist who was aware of the clinical and audiological picture of the patients. Other authors have pointed out that the increase in clinical records using a multicentric collaboration, significantly increases the simultaneous risk of higher variability in imaging criteria and reports^39^.

## Conclusion

Congenital non-syndromic USNHL is frequently linked to inner ear malformations, which occurs, almost always, only on the affected side and is, in most cases, represented by CND combined with CA stenosis. CT and MRI are needed to identify specific abnormalities that could shed light on the pathogenesis of congenital USNHL, affecting the therapeutic approach and the expected benefits of any cochlear implant surgery in about half of infants with non-syndromic congenital USNHL.

## Supplementary Information

Supplementary Table1.

## Data Availability

The datasets generated and analyzed during the current study are available from the corresponding Author on reasonable request.

## Abbreviations

USNHL: Unilateral sensorineural hearing loss
CT: Computed tomography
MR: Magnetic resonance
CND: Cochlear nerve deficiency
IAC: Inner auditory canal
CA: Cochlear aperture
HL: Hearing level
SD: Standard deviation
IP: Incomplete partition
EVA: Enlarged vestibular aqueduct
SCC: Semicircular canal
