# Supplementary material for: Correlation of cochlear aperture stenosis with cochlear nerve deficiency in congenital unilateral hearing loss and prognostic relevance for cochlear implantation
Source: Sci Rep. 2021 Feb 8;11:3338. doi: 10.1038/s41598-021-82818-9 (PMC7870947; doi:10.1038/s41598-021-82818-9)
Supplement: Supplementary file 1 — Supplementary Table1. [file 41598_2021_82818_MOESM1_ESM.docx]

**Correlation of cochlear aperture stenosis with cochlear nerve deficiency in congenital unilateral hearing loss and prognostic relevance for cochlear implantation**

Eva Orzan^1^ MD*, Giulia Pizzamiglio^1^ MD*, Massimo Gregori^2^ MD, Raffaella Marchi^1^ MSc, Lucio Torelli^3^ PhD, Enrico Muzzi^1^ MD

**These authors equally contributed to the study and should be considered first co-authors*

Affiliations:

1Otorhinolaryngology and Audiology, Institute for Maternal and Child Health IRCCS "Burlo Garofolo", Trieste, Italy
2 Radiology, Institute for Maternal and Child Health IRCCS "Burlo Garofolo", Trieste, Italy 3Department of Medicine, Surgery and Health Sciences, University of Trieste, Italy

Corresponding author:

*Giulia Pizzamiglio, MD

Otorhinolaryngology and Audiology, Institute for Maternal and Child Health IRCCS "Burlo Garofolo", Trieste, Italy

e-mail: [giulia.pizzamiglio@burlo.trieste.it](mailto:giulia.pizzamiglio@burlo.trieste.it) , telephone number: +39 3495430512

| *Parameter* | *Range (mm)* | *Affected side (measurement)* | | *Unaffected side (measurement)* | |
| --- | --- | --- | --- | --- | --- |
|  |  |  |  |  |  |
|  |  | Below the range | Above the range | Below the range | Above the range |
| Cochlear Aperture | < 1.2 | 10 | - | 0 | - |
| Basal turn | 8.1-9.7 | 2 | 1 | 6 | 0 |
| Middle turn | 3.8-4.6 | 2 | 7 | 2 | 7 |
| Cochlear height | 3.8-5 | 3 | 0 | 4 | 0 |
| Cochlear length | 8.1-9.6 | 5 | 5 | 5 | 4 |
| Coronal width of the IAC | 3.9-6.7 | 3 | 3 | 0 | 5 |
| Lateral SCC | 2.4-4.8 | 2 | 0 | 2 | 1 |
| Superior SCC | 4.4-6 | 4 | 1 | 2 | 0 |
| Posterior SCC | 4.3-6.3 | 5 | 2 | 3 | 3 |

**Supplement Table S1.** Inner ear measurements ​​that resulted below or above the normal range for the affected and the unaffected side.
